# Viral load and its relationship with the inflammatory response and clinical outcomes in hospitalization of patients with COVID-19

**DOI:** 10.3389/fimmu.2022.1060840

**Published:** 2023-01-04

**Authors:** Mauricio Kuri-Ayache, Andrea Rivera-Cavazos, María Fátima Pérez-Castillo, Juan Enrique Santos-Macías, Arnulfo González-Cantú, José Antonio Luviano-García, Diego Jaime-Villalón, Dalia Gutierrez-González, Maria Elena Romero-Ibarguengoitia

**Affiliations:** ^1^ Cardiology Department, Hospital Clínica Nova de Monterrey, San Nicolás de los Garza, Nuevo León, Mexico; ^2^ Vicerrectoría de Ciencias de la Salud, Escuela de Medicina, Universidad de Monterrey, San Pedro Garza García, Nuevo León, Mexico; ^3^ Research Department, Hospital Clínica Nova de Monterrey, San Nicolás de los Garza, Nuevo León, Mexico; ^4^ Internal Medicine Department, Hospital Clínica Nova de Monterrey, San Nicolás de los Garza, Nuevo León, Mexico; ^5^ Endocrinology Department, Hospital Clínica Nova de Monterrey, San Nicolás de los Garza, Nuevo León, Mexico; ^6^ Infectology Department, Hospital Clínica Nova de Monterrey, San Nicolás de los Garza, Nuevo León, Mexico

**Keywords:** SARS-Cov-2, coronavirus infection, viral load, lactate dehidrogenase, D dimer, interleukein-6, lymphocyte population

## Abstract

**Background:**

The values of viral load in COVID-19 disease have gained relevance, seeking to understand its prognostic value and its behavior in the course of the disease, although there have been no conclusive results. In this study we sought to analyze serum viral load as a predictor of clinical outcome of the disease, as well as its association with inflammatory markers.

**Methods:**

An observational and retrospective study in a private hospital in North Mexico, patients with SARS-COV-2 infection confirmed by reverse transcriptase polymerase chain reaction (RT-PCR) were followed through clinical outcome, viral load measurement, quantification of inflammatory markers and lymphocyte subpopulations. For the analysis, multiple regression models were performed. Results: We studied 105 patients [47 (SD 1.46) years old, 68.6% men]. After analysis with multiple regression models, there was an association between viral load at admission and vaccination schedule (β-value=-0.279, p= 0.007), age (β-value= 0.010, p = 0.050), mechanical ventilation (β-value= 0.872, p = 0.007), lactate dehydrogenase (β-value= 1.712, p= 0.004), D-dimer values at admission (β-value= 0.847, p= 0.013) and subpopulation of B lymphocytes at admission (β-value= -0.527, p= 0.042). There was no association with days of hospitalization, use of nasal prongs or high flux mask. Peak viral load (10 days after symptoms onset) was associated with peak IL-6 (β-value= 0.470, p= 0.011). Peak viral load matched with peak procalcitonin and minimal lymphocyte values. C-reactive protein peak was before the peak of viral load. The minimum value viral load was documented on day 12 after symptom onset; it matched with the minimum values of IL-6 and ferritin, and the peak of D-dimer.

**Conclusions:**

SARS-COV-2 admission viral load is associated with vaccination status, mechanical ventilation, and different inflammatory markers.

## 1 Introduction

The infection caused by SARS-COV-2, the etiological agent of the COVID-19 disease, began to spread from Wuhan, China in December 2019 (1–11), affecting more than 523 million people around the world by May 2022 (12).

The rapid spread of the disease and its high incidence led to high hospital occupancy worldwide (13, 14). In hospitalized patients, the quantification of the serum viral load of SARS-COV-2 through reverse transcriptase polymerase chain reaction (PCR) and its association with other clinical and laboratory parameters became relevant to analyze the progression of viremia and the course of the disease with the aim of identifying ways to predict the clinical outcome of patients (1, 2, 4, 9–11, 13–18) as had been done even in the previous SARS outbreak in the year 2003 (19), however, the results remain controversial.

Multiple studies have sought to identify prognostic tools for clinical outcome such as symptoms, chest radiographs (2) and laboratory findings in blood biometry, C-reactive protein, erythrocyte sedimentation rate, procalcitonin, ferritin, D-dimer and coagulation times (4, 9); yet, they did not include viral load values. Another study showed no significant association between viral load and clinical outcomes, including length of stay, oxygen requirement or survival, however, only non-hospitalized patients were included and only one measurement of viral load levels was taken (11). On the other hand, several cohorts have demonstrated the association between viral load at admission and the risk of developing pneumonia, the severity of the disease, as well as hospital mortality due to COVID-19, especially in older patients and those with medical comorbidities (20, 21). In other cohorts, an association has been demonstrated between high viral load and various cytokines, lactate dehydrogenase and lymphopenia, especially in critically ill patients, and this relationship has also been reported in inflammatory markers such as C-reactive protein (21).

Therefore, we seek to evaluate the role of viral load as a prognostic factor for the clinical outcome in hospitalized patients and its relation to mechanical ventilation requirement,prolongation of hospital stay and mortality. In addition, we sought to identify the relationship of viral load with various inflammatory markers, such as C-reactive protein, lactate dehydrogenase, leukocyte count, lymphocyte count, procalcitonin, ferritin, D-dimer, interleukin 6 (IL-6) and lymphocyte subpopulations, and their behavior during the course of the disease in relation to the time of evolution since the onset of symptoms.

## 2 Materials and methods

### 2.1 Study population and study design

From April 2021 to January 2022, patients with a diagnosis of COVID-19 were evaluated at Hospital Clinica NOVA, a private hospital from Northern Mexico. An observational and retrospective study was performed. The study followed strobe guidelines (22). This study was reviewed and approved by the ethics committee of Universidad de Monterrey with registration number 02-2021-02. Consent form was waived since this is a retrospective study.

The inclusion criteria were hospitalized patients of both genders, adults (18+), confirmed by PCR by nasopharyngeal swab. Patients with previous treatment with antivirals, steroids, convalescent plasma or immunosuppressants were excluded. Also, patients who didn’t had COVID-19 variant, lymphocyte subpopulation and viral load were excluded.

Data such as age, sex, BMI, previous diagnoses of diabetes mellitus, systemic arterial hypertension, chronic kidney disease, COPD, heart disease, among others, were analyzed from the medical chart. The number of days with symptoms, the need for oxygen supplementation on admission, their hospital stays, and vital signs were also recorded to analyze their clinical outcome.

On admission the COVID-19 variant and lymphocyte subpopulation were measured. During the hospital stay, viral load, complete blood count, D-dimer, lactate dehydrogenase, interleukin-6, ferritin and C-reactive protein were taken at least every 24-48 hours. Procalcitonin was measured at admission and in case the patient was suspected to have a secondary bacterial infection. The blood sample was taken through a peripheral venous puncture by the nursing staff.

### 2.2 Sample processing method

#### 2.2.1 Viral load

A peripheral venous blood sample was taken from each patient in a tube with circulating nucleic acid stabilizer (PAXGENE^®^). The samples were transferred at room temperature to an outsource laboratory, PGM Laboratory (Clinical Pathology and Genetics Laboratory) on the same day. They were processed using the circulating nucleic acid extraction kit (QIAGEN^®^) for liquid biopsy and the TaqPath^®^ COVID-19 kit (ThermoFisher Scientific^®^) (23), using a QuantStudio 5 thermal cyclers (Applied Biosystems^®^). The results were sent to our hospital facility and uploaded to the laboratory computer system. The minimum detectable concentration is 10 copies/mL and the maximum is 100,000 copies/mL. Based on these findings and the reports described in the literature, the following reference intervals were defined for results detected in plasma (13): low (< to 100 copies/mL), moderate (>100 to 1000 copies/mL) and high (>1000 copies/mL).

#### 2.2.2 SARS-COV-2 variants

Samples for the study of SARS-COV-2 variants are processed by nucleic acid extraction, then retrotranscription and PCR reaction with a ThermoFisher Veriti endpoint thermal cycler.

#### 2.2.3 Lymphocyte subpopulation

The lymphocyte subpopulation was added, which is extracted by flow cytometry (BD FACS CANTO II IVD, Becton Dikinson, USA) in which the leukocyte count, total lymphocytes, T lymphocytes (CD4 and CD8), B lymphocytes (CD19), NK cells (CD16 and CD56) and CD4/CD8 ratio were analyzed. The antibody used were from Becton Dickinson brand and were as follow: PerCP-Cy 5.5 Anti human CD45, FITC anti human CD3, PE-Cy 7 anti human CD4, APC Cy 7 Anti human CD8, APC Anti human CD 19, PE Anti human CD 16 and PE anti Human CD 56.

### 2.3 Statistical analysis

The distribution of the variables was evaluated with the Shapiro-Wilk test or Kolmogorov and the necessary transformation for normalization was performed. The descriptive analysis of the variables and covariables was performed using parametric statistics with means and standard deviations if they conformed to normality or medians and interquartile ranges otherwise. Multiple regressions were performed to determine the association of viral load with clinical outcomes, and inflammatory response. A density plot was performed between minimum and maximum peak of viral load and inflammatory markers. Missing completely at random values were performed complete case analysis. A value of p < 0.05 was considered significant. Statistical data were analyzed with SPSS R v.4.0.3 vs. 25.

## 3 Results

A total of 571 patients were admitted to the hospital with diagnosis of COVID-19, 105 of those patients had multiple measurements of viral load levels, COVID-19 variant, and lymphocyte subpopulations. From the 105 patients studied, 72 (68.6%) of whom were men with a mean age of 47 years (SD 1.46). Among medical history the most prevalent diseases were obesity in 64 (61%) of the patients with a mean (SD) BMI of 31.67 (1.19). Also, Systemic Arterial Hypertension in 33 (31.4%) and Type 2 Diabetes in 31 (29.5%) of the patients was prevalent. Regarding blood group, 50 (47.6%) of the patients are O+, followed by 23 (21.9%) A+. The demographic data of the patients under study is described in **Table 1**.

**Table 1:** Medical History.

Variable (n=105)	Frequency (%)
Males	72 (68.6)
Smoking	10 (9.5)
Obesity	64 (61.0)
Overweight	28 (26.7)
Systemic arterial hypertension	33 (31.4)
Diabetes mellitus type 2	31 (29.5)
Asthma	7 (6.7)
Ischemic heart disease	7 (6.7)
Heart failure	3 (2.9)
Renal failure	3 (2.9)
Drug allergy	3 (2.9)

Table 1. Shows the medical history of patients hospitalized of SARS-COV-2.

Regarding COVID - 19 vaccines, 35 (33.3%) of the patients had a complete schedule of 2 or more doses and 23 (21.9%) of the patients had an incomplete schedule; 45 (42.9%) of the patients were not vaccinated. Also, 28 (26.7%) of the population was vaccinated with Coronavac and 21 (20%) with ChAdOx1-S. The most frequent SARS-COV-2 variants presented by the patients were Delta in 58 (55.2%), Omicron in 19 (18%), Alpha in 8 (7.6%) and Gamma in 5 (4.8%) patients.

Regarding in-hospital management, nasal prong was used in 71 (67.6%) of patients, 31 (29.5%) required high flow and 11 (10.5%) mechanical ventilation. Three (2%) patients died during follow-up. During the hospital stay, 12 (11.4%) of the patients were admitted to the intensive care unit. Of the 105 patients, 104 (99%) were given apixaban, 99 (94.3%) received baricitinib and 97 (92.4%) remdesivir. Also, 55 (52.4%) of the patients were managed with doxycycline, 57 (54.3%) with ivermectin and 56 (53.3%) with zinc. Patients who had more difficult control with oxygen saturation were given epclusa 23 (21.9%) and/or nitric oxide 11 (10.5%), those with increased IL-6 and in early disease were given tocilizumab 19 (18.1%). In some cases, the use of dexamethasone 18 (17.1%) was necessary (**Table 2**). Patients could receive multiple combination of the drugs.

**Table 2:** In-hospital medication management.

Management	Frequency (%)
Apixaban	104 (99)
Baricitinib	99 (94.3)
Remdesivir	97 (92.4)
Ivermectin	57 (54.3)
Zinc	56 (53.3)
Doxycycline	55 (52.4)
Epclusa	23 (21.9)
Tocilizumab	19 (18.1)
Dexamethasone	18 (17.1)
Nitric oxide	11 (10.5)
Methylprednisolone	4 (3.8)
Convalescent plasma	4 (3.8)
Heparin infusion	4 (3.8)
Vasopressors	4 (3.8)
Enoxaparin	3 (2.9)

Table 2. Shows the medication given during their hospital stay.

Patients could be treated with multiple medications after “hospital stay”.

We calculated a linear regression to predict the effect of viral load at admission on clinical outcome (**Table 3**). The model showed a positive correlation with age (β-value= 0.010, p = 0.050), and mechanical ventilation (β-value= 0.872, p = 0.007), a negative association between the vaccination schedule and viral load (β-value= -0.279, p = 0.007), showing no other relation, such as days of hospitalization, nasal progs, or high flux mask. Mortality was considered initially in our model but was eliminated in the final model since we only had 3 patients that died, and it was difficult to make any correlation with this number of patients.

**Table 3:** Regression model relationship of viral load at admission with prediction of clinical outcome.

	β	Std error	β Std	p-value	95%CI
Constant	1.507	0.27		0	0.97-2.04
Age	0.013	0.005	0.327	0.007	0.004-0.023
Gender	0.189	0.146	0.12	0.201	-0.103-0.48
Vaccination schedule	-0.293	0.102	-0.344	0.005	-0.495
Days of hospitalization	0.006	0.017	0.05	0.723	-0.028- -0.091
Nasal prongs	0.155	0.152	0.099	0.31	-0.147-0.457
High flow	0.058	0.159	0.037	0.715	-0.258-0.374
Mechanical ventilation	0.878	0.317	0.382	0.007	0.248-1.508

Adjusted R-squared= 0.27. Dependent variable: LogViral load at admission.

CI, confidence interval.

Std, standard.

A linear regression was calculated to establish the relationship between viral load at admission and the values of inflammatory markers (lymphocytes, neutrophils, lactate dehydrogenase, C-reactive protein, D-dimer, ferritin and procalcitonin) measured at admission (**Table 4** **).** A positive correlation was found between viral load at admission and lactate dehydrogenase (β-value= 1.712, p= 0.004) and D-dimer values at admission (β-value= 0.847, p= 0.013).

**Table 4:** Regression model of the relationship between viral load at admission and inflammatory markers.

	β	Std error	β Std	p-value	95%CI
Constant	-3.801	2.174		0.085	-8.146-0.544
Lymphocytes on admission	-0.396	0.373	-0.124	0.293	-1.142-0.350
Neutrophils at admission	0.296	0.257	0.125	0.253	-0.217-0.809
Lactate dehydrogenase on admission	1.712	0.574	0.369	0.004	0.565-2.859
C-reactive protein on admission	0.048	0.051	0.107	0.354	-0.055-0.151
D-dimer on admission	0.847	0.331	0.300	0.013	0.185-1.508
Ferritin on admission	-0.186	0.224	-0.108	0.408	-0.634-0.261
Procalcitonin on admission	-0.057	0.149	-0.043	0.702	-0.356-0.241

Adjusted R-squared = 0.226. Dependent variable: Log Viral load at admission

CI, confidence interval.

Std, standard.

Patients could be treated with multiple medications after “hospital stay”.

A linear regression was performed with viral load at hospital admission as the dependent variable, looking for the relationship between it and the vaccination schedule, lactic dehydrogenase, D-dimer, and the subpopulation of B lymphocytes and NK lymphocytes at admission (**Table 5**). A statistically significant positive correlation was found with the values ​​of viral load at admission and D-dimer (β-value= 0.778, p= 0.019). A negative correlation was found between viral load at admission and subpopulation of B lymphocytes at admission (β-value= -0.527, p= 0.042).

**Table 5:** Regression model relationship of viral load at admission with inflammatory markers and lymphocyte subpopulation count at admission.

	β	Std error	β Std	p-value	95%CI
Constant	0.078	1.903		0.968	-3.713 - 3.868
Vaccination schedule	-0.222	0.100	-0.259	0.020	-0.42 2- -0.022
Lactate dehydrogenase on admission	0.669	0.506	0.150	0.190	-0.338 - 1.676
D-dimer on admission	0.778	0.326	0.248	0.019	0.130 - 1.427
Subpopulation of B lymphocytes at admission	-0.527	0.255	-0.239	0.042	-1.034 - -0.020
Subpopulation of natural killer lymphocytes at admission	-0.092	0.282	-0.035	0.746	-0.653 - 0.470

Adjusted R-squared = 0.199. Dependent variable: Log viral load at admission.

CI, confidence interval.

Std, standard.

We calculated a linear regression between the viral load maximum peak during hospital stay and the maximum peak of each of the inflammatory markers (**Table 6**). A positive correlation was found with peak viral load and peak IL-6 (β-value= 0.470, p= 0.011).

**Table 6:** Regression model of the relationship between peak viral load with peak inflammatory markers.

	β	Std error	β Std	p-value	95%CI
Constant	-0.460	2.377		0.847	-5.194 - 4.273
Max. peak leukocytes	-0.364	1.641	-0.083	0.825	-3.631 - 2.902
Max. peak lymphocytes	0.739	0.612	0.145	0.231	-0.480 - 1.957
Max. peak neutrophils	-0.162	1.351	-0.045	0.905	-2.852 - 2.528
Max. peak Lactate dehydrogenase	0.383	0.389	0.125	0.328	-0.391 - 1.157
Max. peak IL-6	0.470	0.180	0.419	0.011	0.112 - 0.827
Max. peak C-reactive protein	-0.266	0.157	-0.180	0.094	-0.579 - 0.047
Max. peak D-dimer	0.310	0.296	0.150	0.300	-0.281 - 0.900
Max. peak ferritin	0.005	0.202	0.003	0.979	-0.397 - 0.408
Max. peak procalcitonin	-0.033	0.123	-0.028	0.788	-0.277 - 0.211

Adjusted R-squared = 0.218. Dependent variable: Log Peak viral load

CI, confidence interval.

Std, standard.

**Figure 1:**
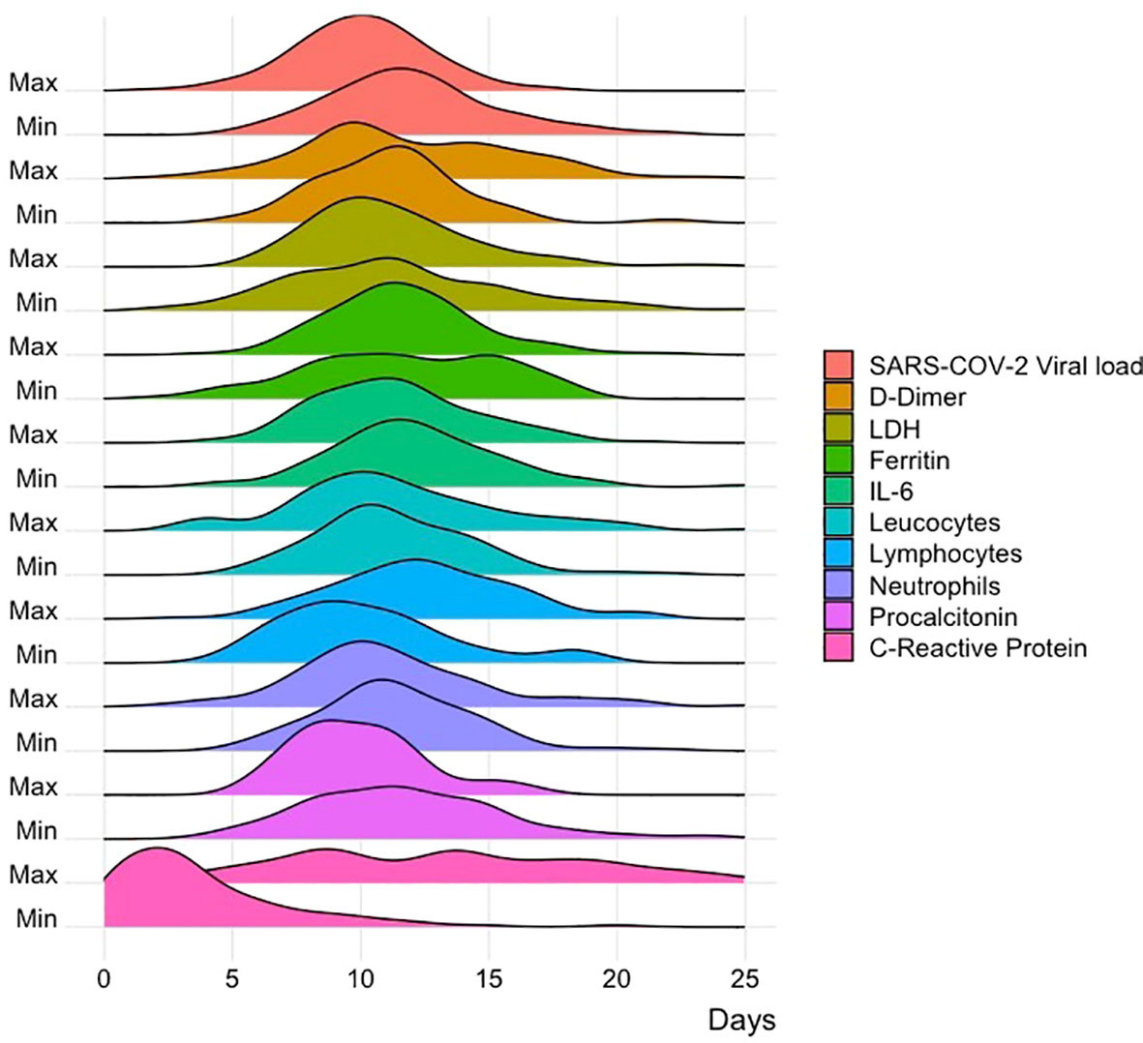
Correlation of viral load with inflammatory markers. Graph correlating the minimum and maximum peak of both viral load and inflammatory markers with the days of evolution of the patients.

A density plot correlating the minimum and maximum peak of both viral load and inflammatory markers with the days of evolution of the patients was made to identify the behavior of viral load and inflammatory marker values during the disease (**Figure 1**). Viral load was found to peak at day 10 after symptom onset, matched with the peak procalcitonin and minimal lymphocyte values. The minimum peak of COVID-19 viral load was documented at day 12 from symptom onset; it matched with the minimum values of IL-6 and ferritin, and the peak of D-dimer. Finally, the peak of C-Reactive Protein was much earlier than viral load peak (around day 3).

## 4 Discussion

In this study, we analyzed the association between COVID-19 viral load in hospitalized patients and its association with clinical outcome and inflammatory markers. Viral load at admission was associated with vaccination status, age, and mechanical ventilation. No association was found between viral load on admission and days of hospitalization, use of oxygen prongs, and high flux mask. Also, a relationship was found between viral load values ​​at admission and D-dimer and lactic dehydrogenase values.

In a retrospective study of 127 patients in Italy, Cocconcelli et al. demonstrated that there is no association between viral load at admission and clinical outcome, including both hospitalized and non-hospitalized patients (2). On the other hand, Dadras et al. in their review article mentioned that there are no conclusive results showing a clear relationship or not between viral load and clinical outcome; it has only been shown that the older the patient, the higher the viral load (1), as we obtained in our data. In contrast to these studies, Boyapati et al. concluded from a study with 1912 patients that viral load was an important determinant for clinical outcome in hospitalized patients requiring supplemental oxygen and ventilation (24). Shenoy in a systematic review (21) of 60 manuscripts concluded that viral load is a predictor of disease and mortality. Kim et al. in a retrospective study comparing the CT scans of 128 patients (21), concluded that viral load may help predict the initial presence of pneumoniae. Our results showed no significant relationship between higher serum viral load and days of hospital stay, and oxygen supplementation. But did find an association with mechanical ventilation. In addition, in our results, patients who were vaccinated had lower viral load levels. An important thing to consider is that our data is from different COVID-19 waves, some causing a more serious condition that might influence when comparing viral load with clinical outcome.

We found association between viral load and inflammatory markers, D-dimer and lactate dehydrogenase at admission were positively correlated with viral load values, this could have a predictive factor for viral load and disease progression, as described in a review article by Porubagheri et al. who list D-dimer as an important marker of disease progression and lactate dehydrogenase as an important marker of lung damage that is elevated in patients with COVID-19 (9). In a study by Qin et al. they concluded that SARS-CoV-2 could act mainly on T lymphocytes, so the study of lymphocyte subpopulations could be useful in the diagnosis and treatment of COVID-19 and described that patients with severe infection had lower values of B, T and NK lymphocytes (26), however, their relationship with viral load had not been studied; when analyzed, we found a negative correlation between B lymphocytes at admission and viral load quantification; however, our results did not show a correlation with NK lymphocyte subpopulations.

It has been established that the peak in viral load values occurs in the first week after the onset of the disease (2), however, its behavior over time in relation to multiple inflammatory markers had not been established. When we analyzed the behavior of inflammatory markers and viral load during the course of the disease, it was found that viral load reaches a peak on day 10 after the onset of symptoms, which coincides with the peak in procalcitonin and the minimum values of lymphocytes, this differs from that described by Cocconcelli et al. who point out that the viral load peak generally occurs in the first week of infection (2), on the other hand, defends the fact that the increase in viral load causes lymphopenia due to lymphocyte depletion, as described by Keam et al. (5) who mentioned it’s associated with greater severity in the disease. Procalcitonin values ​​were not measured daily in the patients, so the result of association between peak viral load and procalcitonin during the course of infection could be biased. On the other hand, the minimum value of viral load coincided with the minimum values of IL-6 and ferritin. Finally, C reactive protein peaked before viral load, this could be an easy and accessible inflammatory marker that predicts the increase of viral load.

The limitations of this study are that the sample only considers hospitalized patients, so we do not know the viral loads of asymptomatic patients or those who received outpatient treatment. Another important limitation to consider when interpreting the results is that our sample is relatively small, which implies that these findings should be validated in larger populations. Likewise, the serum viral load was taken from a peripheral venous blood sample of the patient, where it has been described that nasopharyngeal samples from the lower respiratory tract could be better predictors of clinical outcome and prognosis of patients.

In conclusion, the viral load of SARS-COV-2 is associated with the vaccination status, the age and inflammatory markers such as lactate dehydrogenase, D-dimer, and IL-6. In conclusion, the viral load of SARS-COV-2 is associated with the vaccination status, the age, mechanical ventilation, and inflammatory markers such as lactate dehydrogenase, D-dimer, and IL-6.

## Data availability statement

The database used and analyzed in this study is available from the corresponding author upon reasonable request.

## Ethics statement

The studies involving human participants were reviewed and approved by Ethics committee of Universidad de Monterrey. Written informed consent for participation was not required for this study in accordance with the national legislation and the institutional requirements.

## Author contributions

Conceptualization: MK-A, MR-I, JS-M, JL-G, and DJ-V. Formal analysis: MK-A, AR-C, MP-C, MR-I, and JS-M. Investigation: MK-A, AR-C, MP-C, MR-I, JS-M, JL-G, and DJ-V. Writing – original draft: MK-A, MR-I, and JS-M. Writing – review and editing: MK-A, AR-C, MP-C, MR-I, and JS-M. Project administration: MK-A, MR-I, and JS-M. Supervision: MK-A, MR-I, and JS-M. All authors contributed to the article and approved the submitted version.

## References

[1] Dadras O Afsahi AM Pashaei Z Mojdeganlou H Karimi A Habibi P. The relationship between COVID-19 viral load and disease severity: A systematic review. Immun Inflamm Dis (2022) 10:e580. doi: 10.1002/iid3.580 34904379 PMC8926507

[2] Cocconcelli E Castelli G Onelia F Lavezzo E Giraudo C Bernardinello N. Disease severity and prognosis of SARS-CoV-2 infection in hospitalized patients is not associated with viral load in nasopharyngeal swab. Front Med (2021) 8:714221. doi: 10.3389/fmed.2021.714221 PMC8460755 34568371

[3] Khadke S Ahmed N Ahmed N Ratts R Raju S Gallogly M. Harnessing the immune system to overcome cytokine storm and reduce viral load in COVID-19: a review of the phases of illness and therapeutic agents. Virol J (2020) 17:154. doi: 10.1186/s12985-020-01415-w 33059711 PMC7558250

[4] Samprathi M Jayashree M. Biomarkers in COVID-19: An up-To-Date review. Front Pediatr (2021) 8:607647. doi: 10.3389/fped.2020.607647 33859967 PMC8042162

[5] Keam S Megawati D Patel SK Tiwari R Dhama K Harapan H. Immunopathology and immunotherapeutic strategies in severe acute respiratory syndrome coronavirus 2 infection. Rev Med Virol (2020) 30:e2123. doi: 10.1002/rmv.2123 32648313 PMC7404843

[6] Wan S Yi Q Fan S Lv J Zhang X Guo L. Characteristics of lymphocyte subsets and cytokines in peripheral blood of 123 hospitalized patients with 2019 novel coronavirus pneumonia (NCP). Hematology (2020). doi: 10.1101/2020.02.10.20021832

[7] Fara A Mitrev Z Rosalia RA Assas BM. Cytokine storm and COVID-19: a chronicle of pro-inflammatory cytokines. Open Biol (2020) 10:200160. doi: 10.1098/rsob.200160 32961074 PMC7536084

[8] Pasrija R Naime M. The deregulated immune reaction and cytokines release storm (CRS) in COVID-19 disease. Int Immunopharmacol (2021) 90:107225. doi: 10.1016/j.intimp.2020.107225 33302033 PMC7691139

[9] Pourbagheri-Sigaroodi A Bashash D Fateh F Abolghasemi H. Laboratory findings in COVID-19 diagnosis and prognosis. Clin Chim Acta (2020) 510:475–82. doi: 10.1016/j.cca.2020.08.019 PMC7426219 32798514

[10] Tsukagoshi H Shinoda D Saito M Okayama K Sada M Kimura H. Relationships between viral load and the clinical course of COVID-19. Viruses (2021) 13:304. doi: 10.3390/v13020304 33672005 PMC7919281

[11] Argyropoulos KV Serrano A Hu J Black M Feng X Shen G. Association of initial viral load in severe acute respiratory syndrome coronavirus 2 (SARS-CoV-2) patients with outcome and symptoms. Am J Pathol (2020) 190:1881–7. doi: 10.1016/j.ajpath.2020.07.001 PMC7332909 32628931

[12] COVID-19 map. johns Hopkins coronavirus resour cent (2022). Available at: https://coronavirus.jhu.edu/map.html (Accessed September 20, 2022).

[13] Rodríguez-Serrano DA Roy-Vallejo E Zurita Cruz ND Martín Ramírez A Rodríguez-García SC Arevalillo-Fernández N. Detection of SARS-CoV-2 RNA in serum is associated with increased mortality risk in hospitalized COVID-19 patients. Sci Rep (2021) 11:13134. doi: 10.1038/s41598-021-92497-1 34162948 PMC8222315

[14] Bermejo-Martin JF González-Rivera M Almansa R Micheloud D Tedim AP Domínguez-Gil M. Viral RNA load in plasma is associated with critical illness and a dysregulated host response in COVID-19. Crit Care (2020) 24:691. doi: 10.1186/s13054-020-03398-0 33317616 PMC7734467

[15] Miranda RL Guterres A de Azeredo Lima CH Filho PN Gadelha MR. Misinterpretation of viral load in COVID-19 clinical outcomes. Virus Res (2021) 296:198340. doi: 10.1016/j.virusres.2021.198340 33592214 PMC7881726

[16] Yuan S Jiang S-C Zhang Z-W Fu Y-F Hu J Li Z-L. Quantification of cytokine storms during virus infections. Front Immunol (2021) 12:659419. doi: 10.3389/fimmu.2021.659419 34079547 PMC8165266

[17] Zou L Ruan F Huang M Liang L Huang H Hong Z. SARS-CoV-2 viral load in upper respiratory specimens of infected patients. N Engl J Med (2020) 382:1177–9. doi: 10.1056/NEJMc2001737 PMC7121626 32074444

[18] Liu Y Yan L-M Wan L Xiang T-X Le A Liu J-M. Viral dynamics in mild and severe cases of COVID-19. Lancet Infect Dis (2020) 20:656–7. doi: 10.1016/S1473-3099(20)30232-2 PMC7158902 32199493

[19] Peiris J Chu C Cheng V Chan K Hung I Poon L. Clinical progression and viral load in a community outbreak of coronavirus-associated SARS pneumonia: a prospective study. Lancet (2003) 361:1767–72. doi: 10.1016/S0140-6736(03)13412-5 PMC7112410 12781535

[20] Kim C Kim J-Y Lee EJ Kang YM Song K-H Kim ES. Clinical findings, viral load, and outcomes of COVID-19: Comparison of patients with negative and positive initial chest computed tomography. PloS One (2022) 17:e0264711. doi: 10.1371/journal.pone.0264711 35239734 PMC8893619

[21] Shenoy S. SARS-CoV-2 (COVID-19), viral load and clinical outcomes; lessons learned one year into the pandemic: A systematic review. World J Crit Care Med (2021) 10:132–50. doi: 10.5492/wjccm.v10.i4.132 PMC8291003 34316448

[22] von Elm E Altman DG Egger M Pocock SJ Gøtzsche PC Vandenbroucke JP. The strengthening the reporting of observational studies in epidemiology (STROBE) statement: Guidelines for reporting observational studies. Ann Intern Med (2007) 147:573. doi: 10.7326/0003-4819-147-8-200710160-00010 17938396

[23] Hagman K Hedenstierna M Gille-Johnson P Hammas B Grabbe M Dillner J. Severe acute respiratory syndrome coronavirus 2 RNA in serum as predictor of severe outcome in coronavirus disease 2019: A retrospective cohort study. Clin Infect Dis (2021) 73:e2995–3001. doi: 10.1093/cid/ciaa1285 PMC7499508 32856036

[24] Boyapati A Wipperman MF Ehmann PJ Hamon S Lederer DJ Waldron A. Baseline severe acute respiratory syndrome viral load is associated with coronavirus disease 2019 severity and clinical outcomes: *Post hoc* analyses of a phase 2/3 trial. J Infect Dis (2021) 224:1830–8. doi: 10.1093/infdis/jiab445 PMC8522400 34496013

